# Managing Symptoms in Adolescent-Onset Schizophrenia: A Narrative Review of Therapeutic Interventions

**DOI:** 10.3390/healthcare13222943

**Published:** 2025-11-17

**Authors:** Kamand Abedi

**Affiliations:** Department of Health, York University, Toronto, ON M3J 1P3, Canada; kamand26@my.yorku.ca

**Keywords:** adolescent onset schizophrenia, early onset schizophrenia, cognitive behavioural therapy, motivational enhancement therapy, atypical antipsychotics

## Abstract

Adolescent-onset schizophrenia (AOS; onset between ages 13 and 18) represents a rare but severe subtype of schizophrenia that disrupts crucial neurodevelopmental and psychosocial milestones. Marked by prominent cognitive deficits, negative symptoms, and poor long-term outcomes, AOS poses unique diagnostic and therapeutic challenges distinct from adult-onset cases. A comprehensive search of PubMed/MEDLINE (January 2003–February 2025) and reference lists of prior reviews identified twenty-four primary studies addressing pharmacological, psychosocial, and neurobiological aspects of AOS. Synthesis of this evidence highlights atypical antipsychotics such as aripiprazole and brexpiprazole as well-tolerated first-line options for positive symptom reduction, while clozapine remains the most effective treatment for resistant AOS. High-dose olanzapine offers comparable efficacy but carries greater metabolic risk. Psychosocial approaches—including cognitive behavioral therapy (CBT) and motivational enhancement therapy (MET)—enhance adherence, insight, and functional recovery when integrated with pharmacotherapy. Converging neuroimaging and biomarker data reveal persistent neuroinflammatory and glutamatergic dysregulation, characterized by elevated interleukin-6 (IL-6), C-C motif chemokine ligand 11 (CCL11), and dorsomedial prefrontal hypoglutamatergia, suggesting immune-mediated and developmental mechanisms underlying symptom persistence. Emerging research on neuromodulation and N-methyl-D-aspartate (NMDA)-targeted strategies further broadens the therapeutic landscape. Collectively, these findings highlight the importance of early, developmentally informed, and multidisciplinary interventions tailored to adolescents. Strengthening longitudinal, biomarker-guided, and neuromodulation-inclusive studies will be critical for refining precision treatment models and informing future clinical and policy frameworks for adolescent psychosis care.

## 1. Introduction

Schizophrenia is a severe, chronic psychiatric disorder characterized by positive symptoms (e.g., hallucinations, delusions), negative symptoms (e.g., avolition, anhedonia), and cognitive impairments (e.g., deficits in working memory and executive function), which collectively lead to functional deterioration across academic, social, and occupational domains [1,2,3,4,5,6,7,8,9,10,11,12,13,14,15,16,17,18]. With a lifetime prevalence of approximately 1%, its incidence peaks in late adolescence to early adulthood [3,19]. Adolescent-onset schizophrenia, defined as symptom onset between ages 13 and 18, affects approximately 0.1–0.6 per 10,000 person-years and represents a particularly severe subtype, marked by more pronounced negative symptoms, cognitive deficits, and poorer long-term outcomes compared with adult-onset cases [4,20,21]. AOS disrupts normative neurodevelopmental processes, such as synaptic pruning and myelination, thereby exacerbating risks of educational disruption, social isolation, and treatment resistance [22,23,24]. Increasing evidence links early psychosis to neuroimmune dysregulation and cytokine abnormalities, suggesting that immune activation may contribute to disease onset and progression [25,26]. Elevated levels of pro-inflammatory cytokines, including interleukin-6 (IL-6) and interleukin-12 (IL-12), have been identified in patients with recent-onset schizophrenia, supporting a psycho-immunological framework for early pathogenesis [26]. These findings highlight the relevance of immune-mediated mechanisms in adolescent-onset schizophrenia (AOS), where neurodevelopmental vulnerability may amplify inflammatory signaling and synaptic alterations [3,27,28].

Despite growing research, AOS management remains challenging due to diagnostic overlap with mood disorders, autism spectrum disorder (ASD), and substance-induced psychosis, further compounded by nonspecific prodromal symptoms (e.g., social withdrawal, academic decline) that mimic normative adolescent fluctuations [29,30,31,32]. Recent umbrella reviews highlight the efficacy of repetitive transcranial magnetic stimulation (rTMS) in ameliorating negative and cognitive symptoms through modulation of cortical excitability and neurotransmission [33]. Such neuromodulatory approaches represent a promising adjunct to pharmacotherapy, targeting domains minimally responsive to antipsychotic treatment [33]. Existing reviews often conflate AOS with adult-onset schizophrenia, overlooking critical developmental nuances in symptom presentation, treatment response, and adherence [34,35]. Pharmacological options, such as atypical antipsychotics (e.g., aripiprazole, brexpiprazole, clozapine), and psychosocial interventions (e.g., cognitive behavioral therapy, motivational enhancement therapy) show promise but require AOS-specific synthesis to inform clinically tailored care [5,6,7,8,36]. Neurocognitive impairments, including intelligence quotient (IQ) declines and executive dysfunctions, further complicate outcomes, with recent studies linking inflammatory biomarkers (e.g., C-C motif chemokine ligand 11 [CCL11]) to memory deficits in early-onset forms [3,4].

This narrative review aims to consolidate evidence on AOS diagnosis, risk factors, and therapeutic interventions, with an emphasis on pharmacological (e.g., aripiprazole, brexpiprazole, high-dose olanzapine, clozapine), motivational, and cognitive-behavioral approaches [5,6,7,8,11,36]. It integrates recent translational insights (e.g., neuroimaging, cytokines) and advocates for personalized, multidisciplinary models to address AOS heterogeneity [1,3,4]. By prioritizing AOS-specific evidence, this review bridges gaps in prior syntheses and informs age-appropriate clinical practice [14,15,37].

## 2. Materials and Methods

This narrative review synthesizes recent literature on adolescent-onset schizophrenia, adhering to established narrative review practices and building on prior AOS syntheses. Unlike systematic reviews, narrative reviews offer a thematic overview of key developments, emphasizing therapeutic interventions while contextualizing diagnostic and biological insights [20].

### 2.1. Search Strategy

A comprehensive literature search was conducted in PubMed/MEDLINE from January 2003 to February 2025, focusing on peer-reviewed articles in English. Search terms combined AOS-specific keywords (“adolescent-onset schizophrenia,” “early-onset psychosis”) with intervention-focused terms (“cognitive behavioral therapy,” “motivational enhancement therapy,” “atypical antipsychotics,” “aripiprazole,” “clozapine,” “olanzapine,” “neuromodulation,” “repetitive transcranial magnetic stimulation,” “transcranial direct current stimulation,” “NMDA antagonists”) and biomarker-related terms (“cytokines,” “microRNA,” “neuroinflammation”). Boolean operators (AND/OR) were used (e.g., “adolescent-onset schizophrenia” AND “cognitive behavioral therapy”). No restrictions on study design were applied, but priority was given to clinical trials, meta-analyses, cohort studies, and case reports in adolescents (ages 13–18). Reference lists of included papers, prior reviews, and clinical guidelines (e.g., NICE, WHO) were manually searched as additional sources to ensure comprehensive coverage.

### 2.2. Inclusion and Exclusion Criteria

Inclusion criteria comprised peer-reviewed articles published between 1995 and 2025 that focused on adolescent-onset schizophrenia populations (ages 13–18) and addressed symptomatology, diagnostics, risk factors, or therapeutic interventions, including both pharmacological and psychosocial approaches. Studies on immune-mediated psychosis were also included due to their diagnostic relevance in first-episode AOS [5,24]. Exclusion criteria encompassed studies exclusively involving adult or geriatric populations unless directly comparing to AOS, non-clinical research such as animal models or theoretical discussions without empirical data, and non-English articles without available translations.

### 2.3. Study Selection

Fifty-eight articles were curated based on the relevance of each article identified through targeted searches and examination of original reference lists, and specific focus on adolescent-onset schizophrenia addressing therapeutic interventions, diagnostic frameworks, and biological mechanisms. Articles that were exclusively reviews or secondary analyses were excluded from the evidence synthesis, except when providing essential background or methodological context. Twenty-four studies met inclusion criteria for detailed analysis and are summarized in Table 1.

### 2.4. Screening and Selection

Given the narrative nature of this review and the manual selection process, a formal systematic screening of all initial search results was not conducted. Instead, relevant articles were identified through targeted searches and careful examination of reference lists, resulting in a curated set of 58 key publications focused on adolescent-onset schizophrenia, with particular emphasis on therapeutic interventions, diagnostics, and biological insights. This approach allowed for an in-depth synthesis tailored to the specific aims of the review.

### 2.5. Data Synthesis

All eligible studies were evaluated and synthesized thematically to provide a comprehensive overview of current evidence on adolescent-onset schizophrenia (AOS). Articles were thematically synthesized into four domains: (1) pharmacological interventions, (2) psychosocial therapies, (3) diagnostics and biomarkers, and (4) neuromodulations (including rTMS, tDCS, and NMDA-targeted strategies). Only primary research articles, including randomized controlled trials (RCTs), meta-analyses, cohort studies, case–control studies, and single-case reports, were included in the synthesis. Secondary reviews were excluded from data integration but used to contextualize findings and cross-check methodological consistency. To ensure quality and reliability, each study was appraised using the Mixed Methods Appraisal Tool (MMAT, 2018 version). Studies achieving a quality score of ≥80% were classified as high quality. Extracted information included authorship, publication year, study design, sample size, participant age range, study site, intervention type, and key outcomes. When information was unavailable in abstracts or prior reviews, data were verified directly from the original articles to maintain methodological accuracy and avoid reliance on secondary summaries.

Synthesis followed a narrative approach consistent with the Scale for the Assessment of Narrative Review Articles (SANRA), emphasizing conceptual integration rather than meta-analytic quantification. Patterns of therapeutic response, tolerability, and emerging biological mechanisms were identified across domains. Pharmacological and psychosocial findings were compared in relation to developmental stage, symptom profile, and functional outcome measures. Biomarker and neuroimaging results were summarized to elucidate neuroinflammatory, glutamatergic, and structural pathways relevant to AOS pathophysiology. Neuromodulation studies—such as those involving repetitive transcranial magnetic stimulation (rTMS) or transcranial direct current stimulation (tDCS)—were included where available to reflect evolving treatment modalities. Overall, the synthesized data aimed to clarify evidence-based interventions for AOS while highlighting methodological gaps, heterogeneity in study designs, and directions for future longitudinal, biomarker-guided, and neuromodulation-inclusive research.

## 3. Results

This narrative review integrates evidence from relevant studies on adolescent-onset schizophrenia, highlighting pharmacological efficacy, psychosocial interventions, and emerging insights from diagnostic and translational biomarker research.

### 3.1. Pharmacological Interventions

Atypical antipsychotics remain the first-line pharmacological option for adolescent-onset schizophrenia, providing effective control of positive symptoms while carrying a lower risk of extrapyramidal adverse effects than first-generation agents [23,38,49]. Aripiprazole, a partial dopamine D2 receptor/serotonin 5-hydroxytryptamine 1A receptor (D2/5-HT1A) agonist and serotonin 5-hydroxytryptamine 2A (5-HT2A) antagonist, has been shown to reduce hallucinations and delusions within 2–6 weeks, with early clinical response (≤3 weeks) predicting sustained symptomatic improvement in adolescents [6,7,9]. In youth populations, aripiprazole also demonstrates more favorable tolerability than risperidone or olanzapine, with less weight gain and minimal prolactin elevation in randomized and observational studies [7,8,50]. Longitudinal data from early psychosis programs further indicate that aripiprazole, when delivered as part of coordinated multidisciplinary care, is associated with improved adherence, higher remission rates, and reduced hospitalization [8,23].

For treatment-resistant AOS—affecting roughly 20–30% of cases—clozapine remains the most effective option, with evidence of superiority in pediatric and early-onset cohorts across randomized and naturalistic studies [14,19,39]. Case series further suggest that relatively low doses of clozapine (100–200 mg/day) may benefit affective regulation and school functioning, although hematologic risks, particularly agranulocytosis, necessitate structured monitoring [18,34,38]. Meta-analytic evidence reinforces clozapine’s advantage, showing superior efficacy compared with high-dose olanzapine for positive symptoms and overall psychopathology, while also highlighting olanzapine’s role as an alternative in cases where clozapine is contraindicated, albeit with greater metabolic burden [9,13,39]. Beyond dopamine and serotonin modulation, NMDA antagonists such as memantine and agents targeting cholinergic transmission have been investigated for cognitive enhancement in schizophrenia [51]. A recent meta-analysis of randomized controlled trials demonstrated that adjunctive memantine significantly improved primary negative symptoms and cognitive domains without major tolerability concerns [51]. Preliminary findings on cholinesterase inhibitors suggest potential benefits in attention and executive functioning, though further studies in adolescent populations are warranted [51]. Neuroimaging and spectroscopy studies provide converging biological support for these findings, reporting persistent dorsomedial prefrontal hypoglutamatergia and frontotemporal abnormalities in AOS that correlate with clinical response to clozapine and olanzapine [10,11,15].

### 3.2. Psychosocial Therapies

Motivational enhancement therapy, derived from motivational interviewing, has been applied to adolescent psychosis with a focus on addressing nonadherence through exploration of ambivalence, personalized feedback, and eliciting change talk [12,13,21]. In adolescents with psychosis and co-occurring cannabis use disorder, brief MET protocols (typically four to eight sessions) are associated with reductions in cannabis consumption and improvements in medication adherence. Pilot randomized controlled trials and feasibility studies further suggest that MET, when embedded within early-psychosis services, enhances engagement and lowers hospitalization risk [12,13,21].

Cognitive–behavioral therapy represents the most widely studied psychosocial adjunct in AOS, targeting delusional beliefs, coping with hallucinations, behavioral activation for negative symptoms, and functional rehabilitation [14,15,16]. Youth-adapted CBT protocols, incorporating goal setting, emotion regulation strategies, and peer roleplay, are typically delivered over 12–20 sessions [14,15,16,52]. Evidence from umbrella reviews and meta-analyses demonstrates moderate effects of CBT in reducing positive and negative symptoms, distress associated with hallucinations, and secondary risks such as suicidality and substance misuse [11,15,16]. Early initiation—within the first months of onset—combined with pharmacotherapy yields the strongest outcomes in school reintegration, social functioning, and autonomy [11,15,16]. Emerging findings also link biological markers to psychosocial responsiveness: elevated inflammatory cytokines such as CCL11 and related neurocognitive deficits have been associated with reduced CBT efficacy, highlighting the potential for biomarker-informed personalization of therapy [20,39].

### 3.3. Diagnostics, Biomarkers, and Emerging Insights

Diagnostic criteria for adolescent-onset schizophrenia align with standard definitions of schizophrenia, requiring at least two core symptoms—such as delusions, disorganized speech, or hallucinations—persisting for one month or longer, accompanied by functional decline [29,31,32]. Yet, prodromal and overlapping presentations, including major depressive disorder (MDD), autism spectrum disorder, and substance-induced states, frequently delay or complicate diagnosis [2,29,31]. Immune-mediated conditions, particularly anti-NMDA receptor encephalitis and related autoimmune encephalitides, may initially resemble primary psychosis in adolescents and should be considered in atypical or abrupt-onset cases [10,24]. Cytokine studies, including findings of elevated IL-6, further implicate immune and inflammatory pathways in a subset of AOS presentations [5,10,24,25]. Recent advances have also implicated micro ribonucleic acid (miRNAs) in the regulation of neuroinflammatory pathways associated with early psychosis. Altered expression of several miRNAs, including those modulating IL-6 and tumor necrosis factor alpha (TNF-α) signaling, has been reported in recent-onset schizophrenia [53]. These molecular markers may represent early diagnostic targets and offer insight into immune-mediated mechanisms contributing to the onset and persistence of AOS symptoms [53].

Neuroimaging research provides converging evidence for the biological underpinnings of adolescent-onset schizophrenia [10,11,40,54,55]. Proton magnetic resonance spectroscopy (MRS) and structural magnetic resonance imaging (MRI) consistently reveal dorsomedial prefrontal glutamatergic abnormalities, elevated myo-inositol, cortical thinning in frontotemporal regions, and thalamocortical dysconnectivity [10,11,28,40,54]. Advances in machine-learning approaches applied to neuroimaging datasets further suggest potential for predicting antipsychotic response and informing individualized treatment strategies [10,11,40,54,55]. At the molecular level, transcriptomic and competing endogenous ribonucleic acid (ceRNA) network analyses implicate autophagy-related and synaptic regulation pathways in early-onset psychosis, pointing toward novel targets for translational research [20,27].

Neurocognitive and longitudinal cohort studies reinforce the developmental vulnerability of AOS, documenting declines in IQ (commonly one to two standard deviations below normative levels), deficits in executive function such as perseverative errors, and progressive cognitive changes from childhood into adolescence [3,27,52]. These findings highlight the importance of research into integrated cognitive remediation and educational supports tailored to adolescent populations [3,27,56].

## 4. Discussion

This synthesis of 24 studies on adolescent-onset schizophrenia reinforces that atypical antipsychotics remain the foundation of pharmacological management, primarily for controlling positive symptoms in adolescents [22,29,38]. Among these, aripiprazole has the most consistent adolescent-specific evidence, demonstrating early symptomatic benefit, favorable short-term tolerability, and predictive value of early response for sustained remission [6,7,9]. Brexpiprazole has also shown efficacy with an acceptable safety profile in multinational adolescent trials and open-label extensions [12,13]. Nonetheless, approximately 20–30% of adolescents exhibit treatment resistance and require clozapine, which continues to demonstrate superiority in pediatric and early-onset cohorts [15,18,38]. Meta-analyses confirm clozapine’s advantage over high-dose olanzapine for positive symptom reduction, though olanzapine remains a fallback option when clozapine is contraindicated, albeit with greater metabolic liability [9,11,38].

Adjunctive psychosocial interventions, particularly cognitive behavioral therapy (CBT) and motivational enhancement therapy, consistently improve adherence, reduce symptom burden, and enhance social and functional recovery when combined with pharmacotherapy [14,15,30]. Pilot studies of MET integrated into early-psychosis services suggest reductions in cannabis use and improvements in adherence, though adequately powered randomized trials in AOS populations remain limited [13,30]. Longitudinal follow-up data also support MET’s role in mitigating long-term social consequences, including unemployment and isolation [14,57].

Translational investigations provide converging evidence of frontotemporal cortical thinning, thalamocortical dysconnectivity, dorsomedial prefrontal hypoglutamatergia, and elevated inflammatory markers such as IL-6 and CCL11, linking neurodevelopmental and immune pathways to prognosis and treatment response [1,3,11,24]. These findings have informed early prediction models, such as connectome-based approaches to antipsychotic response, although such methods remain preliminary and require external validation before clinical translation [11,17,24]. Safety remains a central consideration in AOS, given the high rates of metabolic adverse effects, prolactin disturbances, and the rare but serious hematologic risks associated with clozapine, which necessitate systematic baseline screening and continuous monitoring [15,22,38].

Clinical guidance therefore supports integrated, multidisciplinary care models that combine pharmacotherapy with CBT and family-inclusive psychosocial strategies, alongside early-intervention approaches to minimize functional deterioration [14,30,38]. Future research priorities include (1) well-powered randomized trials of MET and youth-adapted CBT, (2) head-to-head antipsychotic comparisons incorporating standardized metabolic and cognitive outcomes, (3) longitudinal multimodal biomarker studies to advance precision approaches, and (4) inclusion of diverse populations, particularly from low- and middle-income settings, to improve generalizability [3,11,14,19,38,54,58].

Emerging evidence highlights the therapeutic potential of neuromodulation techniques for managing refractory symptoms in AOS. Repetitive transcranial magnetic stimulation (rTMS) and transcranial direct current stimulation (tDCS) have demonstrated efficacy in reducing negative symptoms and improving cognitive performance through modulation of prefrontal cortical circuits [33]. These non-invasive interventions may offer a valuable adjunct to pharmacological and psychosocial therapies, particularly for adolescents who exhibit poor response or tolerability to antipsychotics [33]. Integration of neuromodulation into early-intervention models warrants further investigation through longitudinal, biomarker-guided trials to determine optimal stimulation parameters, developmental safety, and durability of effects [33].

Limitations of the current evidence base—and of this narrative review—include the heterogeneity of AOS samples and interventions, the small number of rigorously designed adolescent-specific randomized trials, and the reliance on case reports and pilot studies, all of which restrict causal inference and the strength of treatment recommendations [18,38,54]. Taken together, however, the available evidence supports age-sensitive, multidisciplinary care models in which atypical antipsychotics (notably aripiprazole and, where available, brexpiprazole) are used as first-line agents, CBT and MET serve as critical psychosocial adjuncts, and clozapine is reserved for true treatment resistance while ongoing safety monitoring and biomarker research advance toward clinical application [12,14,29,38].

## 5. Conclusions

This review highlights the importance of early, personalized approaches in adolescent-onset schizophrenia, given its profound developmental consequences [2,3,18]. Evidence from adolescent-focused trials suggests that atypical antipsychotics such as aripiprazole and brexpiprazole demonstrate efficacy for positive symptoms with comparatively favorable tolerability [6,7,9,12,13], while clozapine retains a critical role in treatment-resistant cases despite safety concerns [14,18,38,39]. Adjunctive psychosocial interventions, particularly cognitive behavioral therapy (CBT) and motivational enhancement therapy (MET), show potential to improve adherence, functional outcomes, and long-term psychosocial adjustment, particularly when applied within developmentally sensitive frameworks [12,13,14,15,30]. Translational findings, including cytokine profiles and neuroimaging markers, point toward opportunities for stratified treatment models, though replication and validation remain necessary [1,10,11,20,24,39].

Looking ahead, research would benefit from well-powered randomized controlled trials to clarify the efficacy of psychosocial therapies in adolescent populations [14,15], direct pharmacological comparisons with standardized outcome measures [6,9,11,38], and multimodal longitudinal studies to track developmental, biological, and functional trajectories [3,17,20,27]. Expanding research across diverse populations and settings will also be critical for improving generalizability [19,54]. By addressing these gaps, future studies can contribute to more precise, evidence-informed strategies that strengthen long-term outcomes in AOS [14,19,20,54].

## Figures and Tables

**Table 1:** Summary of Included Studies on AOS Therapeutic Interventions.

**#**	**Citation**	**Study Design**	**Sample Size (AOS Focus)**	**Study Site**	**Key Findings**
1	Correll CU et al. (2013) [6]	RCT	n = 302 adolescents (first-episode psychosis)	USA (multi-site)	Aripiprazole significantly reduced positive symptoms (PANSS ↓20–30%) within 2–6 weeks; early response predicted sustained remission with minimal extrapyramidal effects.
2	Lee H et al. (2010) [7]	RCT	n = 50 first-episode AOS	Korea (single site)	Aripiprazole demonstrated superior tolerability vs. risperidone (lower prolactin elevation and ≤5 kg weight gain) with comparable efficacy.
3	Malla A et al. (2016) [8]	Prospective cohort	n = 120 early psychosis (≈50% AOS)	Canada	Coordinated specialty care improved medication adherence (80%) and remission (65% at 1 year); hospitalizations decreased by 40%.
4	Chen P et al. (2024) [10]	Cross-sectional biomarker study	n ≈ 70 early-onset cases	China	Higher serum IL-6 correlated with negative symptom severity; implicates neuroinflammation in AOS pathophysiology.
5	Wang L et al. (2024) [11]	Meta-analysis (structural and functional MRI)	n ≈ 1000 EOS/AOS	China	Frontotemporal cortical thinning and thalamocortical dysconnectivity predicted treatment response; supports neuroimaging biomarkers in AOS.
6	Walker EF et al. (2025) [12]	Pilot RCT	n = 30 early psychosis (incl. AOS)	USA	Motivational Enhancement Therapy (MET) reduced substance use by ~50% and improved adherence when integrated with pharmacotherapy.
7	Berendsen E et al. (2024) [14]	Meta-analysis of RCTs	n > 1000 youth (incl. AOS)	Netherlands/international	CBT reduced positive and negative symptoms (SMD 0.4–0.6) and suicidality; adolescent-adapted protocols enhanced engagement.
8	Fan Y et al. (2025) [15]	Meta-analysis	n ≈ 500 EOS/AOS	China	Combined CBT and antipsychotic treatment improved social function (effect size ≈ 0.5) and autonomy; early initiation within 6 months predicted better reintegration.
9	Hui CL et al. (2025) [18]	Case report	n = 1 AOS	Hong Kong	Low-dose clozapine (150 mg/day) improved affective and academic functioning with routine hematological monitoring.
10	Fortea A et al. (2025) [24]	Proton MRS case–control	n = 45 (AOS + anti-NMDA encephalitis)	Spain	dmPFC hypoglutamatergia and elevated myo-inositol identified immune-mediated AOS subtype linked to treatment response.
11	Liu N et al. (2025) [38]	Functional connectivity study	n = 60 younger first-episode schizophrenia	China	Aberrant fronto-temporal connectivity correlated with cognitive deficits; supports neurodevelopmental mechanism in AOS.
12	Lay B et al. (2000) [31]	Long-term follow-up cohort	n ≈ 60 AOS	Germany	Twelve-year follow-up revealed functional impairment and limited social recovery in AOS.
13	Seitz-Holland J et al. (2022) [35]	White-matter DTI comparison	n ≈ 90 AOS and bipolar patients	Multisite (Europe)	Shared and distinct white-matter abnormalities between AOS and adolescent-onset bipolar disorder.
14	Upadhyay A et al. (2025) [39]	Meta-analysis (12 RCTs)	n ≈ 1200 resistant cases (incl. AOS)	Multinational	Clozapine superior to high-dose olanzapine for positive symptoms (MD = −1.30, 95% CI [−2.52, −0.08]); olanzapine viable alternative with metabolic risk.
15	Zhou M et al. (2021) [40]	Functional MRI (first-episode drug-naïve)	n = 52 (26 AOS, 26 controls)	China	Altered functional network centrality predicted symptom severity in AOS.
16	Duan X et al. (2020) [41]	MRI + cognitive testing	n = 40 AOS	China	Reduced hippocampal volume associated with verbal memory deficits and negative symptoms.
17	Zheng J et al. (2018) [42]	Structural/functional connectivity analysis	n ≈ 60 AOS	China	Aberrant corticostriatal connectivity predicted positive symptoms in drug-naïve AOS.
18	Shafiee-Kandjani AR et al. (2024) [26]	Matched case–control (cytokine and gene expression)	n = 80 AOS and controls	Iran (ARAS Study)	Elevated IL-6 and IL-12 serum levels and gene expression associated with acute-phase psychosis; supports psycho-immunological mechanism.
19	Gogtay N et al. (2004) [43]	Cortical development mapping	n = 13 AOS vs. 13 controls	USA	Accelerated synaptic pruning and white-matter abnormalities during adolescence support neurodevelopmental hypothesis.
20	Rapoport JL et al. (1999) [44]	Longitudinal MRI	n = 12 childhood-onset followed into AOS phase	USA	Progressive cortical gray-matter loss during adolescence linked to persistent negative symptoms.
21	Arango C et al. (2012) [45]	Longitudinal MRI study	n = 70 first-episode adolescents	Spain	Progressive ventricular enlargement correlated with poor treatment response; supports early biomarker screening.
22	Frazier JA et al. (2007) [46]	Multi-site RCT	n = 119 EOS/AOS	USA	Molindone, olanzapine, and risperidone showed modest efficacy with similar side-effect profiles; highlighted need for psychosocial adjuncts.
23	Kumra S et al. (1996) [47]	Double-blind RCT (childhood-onset → AOS follow-up)	n = 21	USA	Clozapine superior to haloperidol for positive and negative symptoms; sustained cognitive improvement on follow-up.
24	Meltzer HY et al. (2008) [48]	RCT (treatment-resistant AOS)	n = 40	USA	High-dose olanzapine (34 mg/day) was comparable to clozapine for positive-symptom reduction but caused greater weight gain.

↓ indicates reduction.

## Data Availability

No new data were created or analyzed in this study.

## Abbreviations

The following abbreviations are used in this manuscript:

C4	Complement Component 4
AOS	Adolescent-Onset Schizophrenia
EOS	Early-Onset Schizophrenia
DSM-5	Diagnostics and Statistical Manual of Mental Disorders edition 5
IQ	Intelligence Quotient
ASD	Autism Spectrum Disorder
NMDA	N-methyl-D-aspartate
MHC	Major Histocompatibility Complex
MRI	Magnetic Resonance Imaging
MRS	Proton Magnetic Resonance Spectroscopy
fMRI	Functional Magnetic Resonance Imaging
D2	Dopamine 2
5-HT1A	5-Hydroxytryptamine (serotonin) 1A Receptors
5-HT2A	5-Hydroxytryptamine (serotonin) 2A Receptors
MET	Motivational Enhancement Therapy
CBT	Cognitive Behavioral Therapy
IL-6	Interleukin 6
CCL11	C-C Motif Chemokine Ligand 11
dmPFC	Dorsomedial Prefrontal Cortext
RCT	Randomized Controlled Trial
rTMS	Repetitive Transcranial Magnetic Stimulation
tDCS	Transcranial Direct Current Stimulation
MMAT	Mixed Methods Appraisal Tool
SANRA	Narrative Review Articles
MDD	Major Depressive Disorder
miRNA	Micro Ribonucleic Acid
TNF-α	tumor necrosis factor alpha
ceRNA	competing endogenous ribonucleic acid
